# Supplementary material for: Hydrolysis-deficient mosaic microtubules as faithful mimics of the GTP cap
Source: Nat Commun. 2025 Mar 10;16:2396. doi: 10.1038/s41467-025-57555-6 (PMC11893814; doi:10.1038/s41467-025-57555-6)
Supplement: Supplementary file 1 — Supplementary information [file 41467_2025_57555_MOESM1_ESM.pdf]

Supplemental information

Hydrolysis-deficient mosaic microtubules as faithful mimics of the GTP cap

Juan Estévez-Gallego*, Thorsten B. Blum+, Felix Ruhnow+, María Gili, Silvia Speroni, Raquel García-Castellanos, Michel O. Steinmetz and Thomas Surrey*.

* Corresponding authors: juan.estevez-gallego@psi.ch, thomas.surrey@crg.eu.
+ Contributed equally

This PDF file includes:

- Supplementary figures 1 to 9
- Supplementary tables 1 to 3

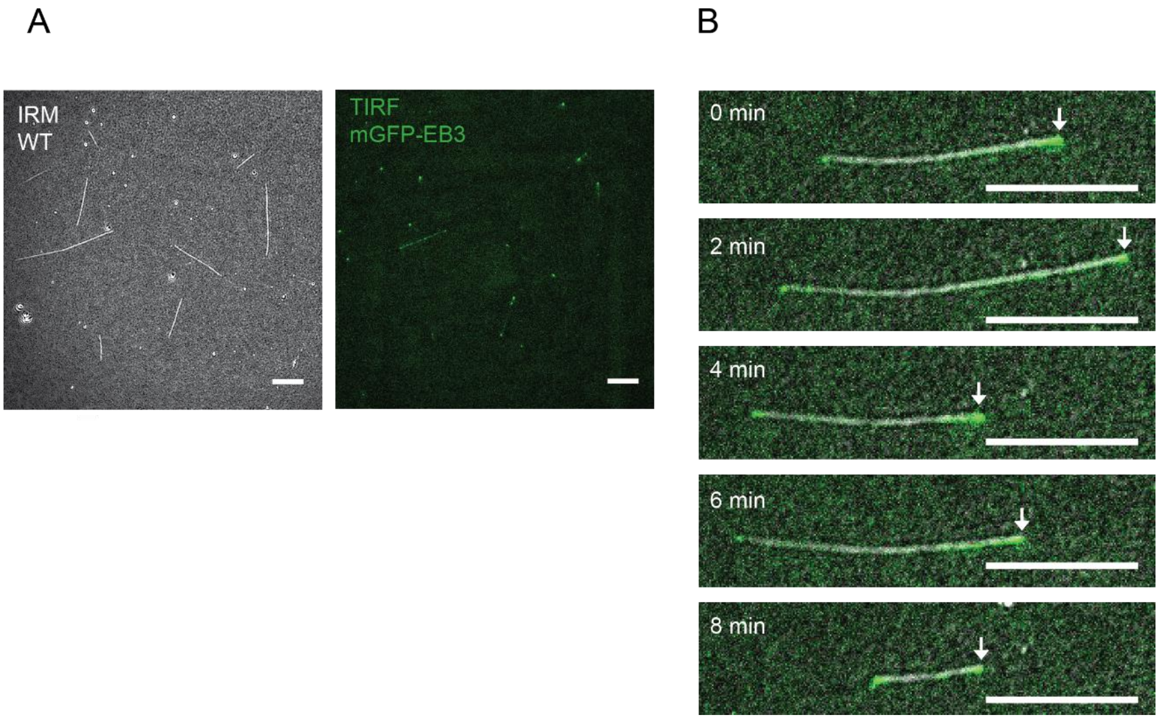

Supplementary Figure 1. mGFP-EB3 binding to wild type human microtubules. A. IRM/TIRF microscopy images of unlabeled E254E (WT) microtubules, elongating from glass-attached GMPCPP-microtubule seeds in the presence of 40 nM mGFP-EB3. **B.** Magnified view of a single microtubule at different times, as indicated. White arrows highlight the EB3 comet at the growing microtubule plus end. The microtubule displays dynamic instability, switching between growth and shrinkage; all images show the microtubule in a state of growth. Scale bars represent 10 µm.

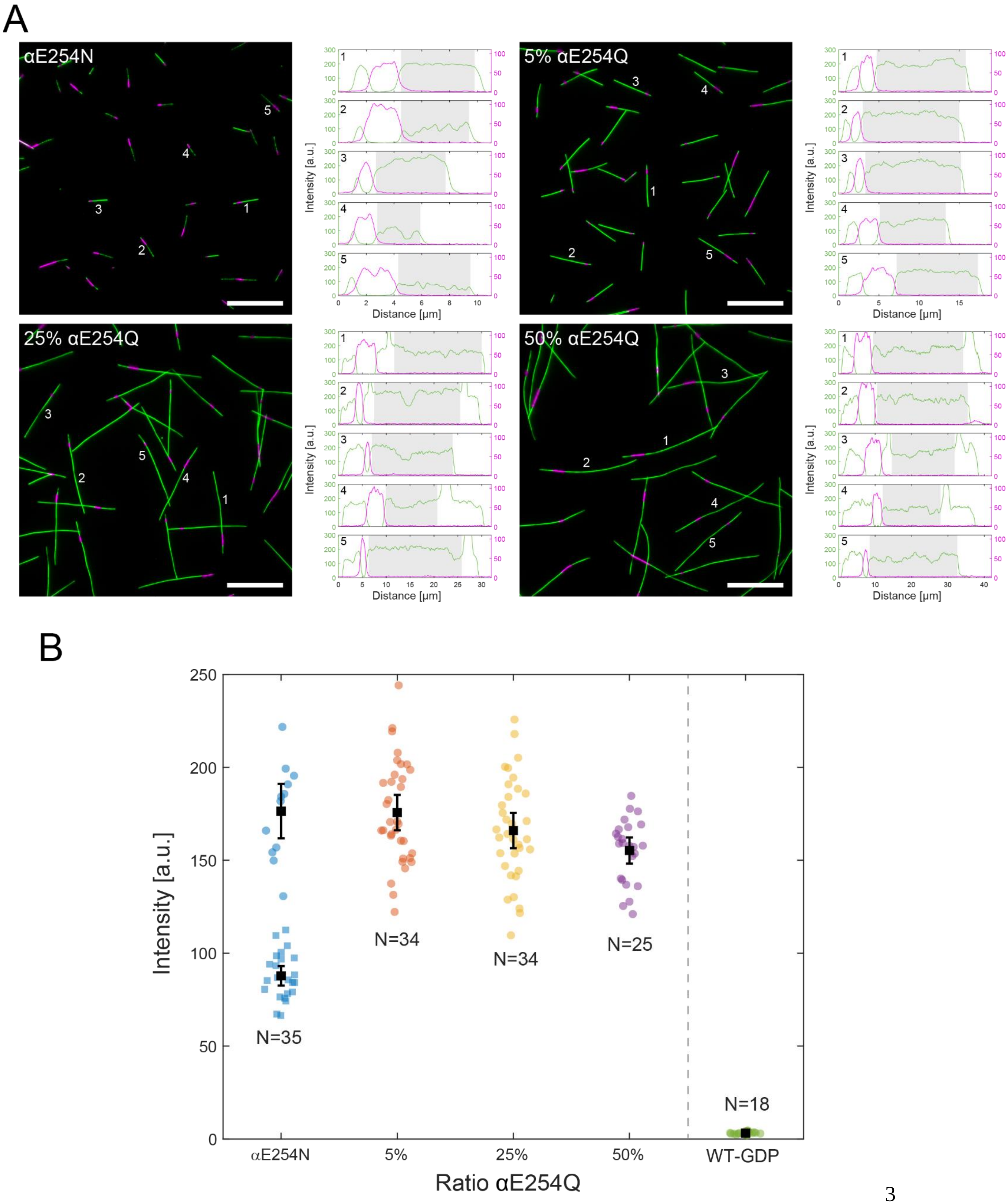
A
αE254N
5% αE254Q
25% αE254Q
50% αE254Q
Intensity [a.u.]
Distance [µm]
B
Intensity [a.u.]
N=35
N=34
N=34
N=25
N=18
αE254N
5%
25%
50%
WT-GDP
Ratio αE254Q

Supplementary Figure 2. Quantitative analysis of the mGFP-EB3 intensity along different microtubules. A. TIRF microscopy images (same contrast for all 4 images; scale bar 20 µm) of mGFP-EB3 (green) binding to αE254N-microtubules and to αE254Q+αE254N mosaic microtubules at the indicated αE254Q percentages in the tubulin mixture, elongated from GMPCPP-microtubule seeds (magenta). Line scans show integrated intensity of mGFP-EB3 (green) and GMPCPP-seeds (magenta). Gray area indicates intensities used for averaging the EB3-GFP signal along every microtubule plus segment (excluding seeds and microtubule crossings). **B.** Average mGFP-EB3 intensities for conditions shown in A and for wild type GDP-microtubule segments (excluding the GTP cap region). N = number of microtubules analyzed for each condition. For αE254N-microtubules, we could manually classify microtubules with brighter (circles, n=12) and dimmer (squares, n=23) mGFP-EB3 coverage. The total tubulin concentration was always 10 µM. Error bars indicate mean ± 2*SEM (95%CI).

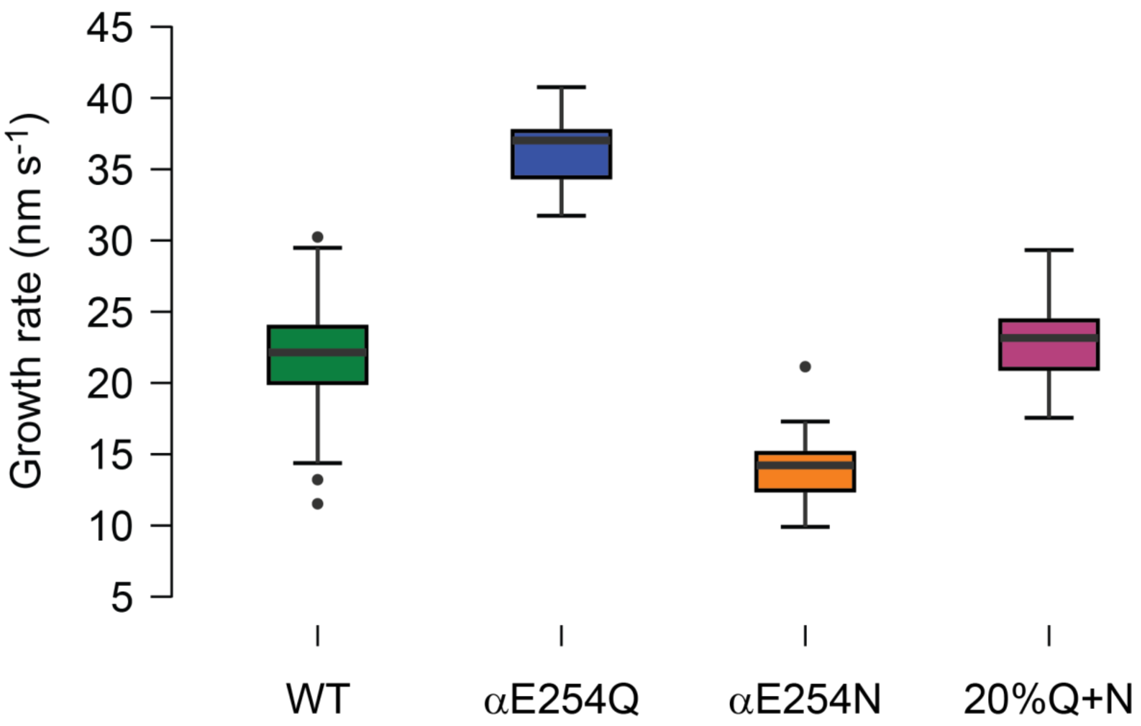

Supplementary Figure 3. Comparison of microtubule growth rates. Box plots of microtubule growth rates at a total concentration of 10 µM of WT (green), αE254Q (blue), αE254N (orange), and 20%Q+N-tubulin (purple). Within each box, horizontal black lines denote median values; boxes extend from the 25th to the 75th percentile of each group's distribution of values; vertical extending lines denote the extreme values within 1.5 interquartile range of the 25th and 75th percentile of each group; dots denote observations outside the range of adjacent values. These data are the same as those shown in Figure 4B and Supplementary Table 1 for the 10 µM tubulin condition.

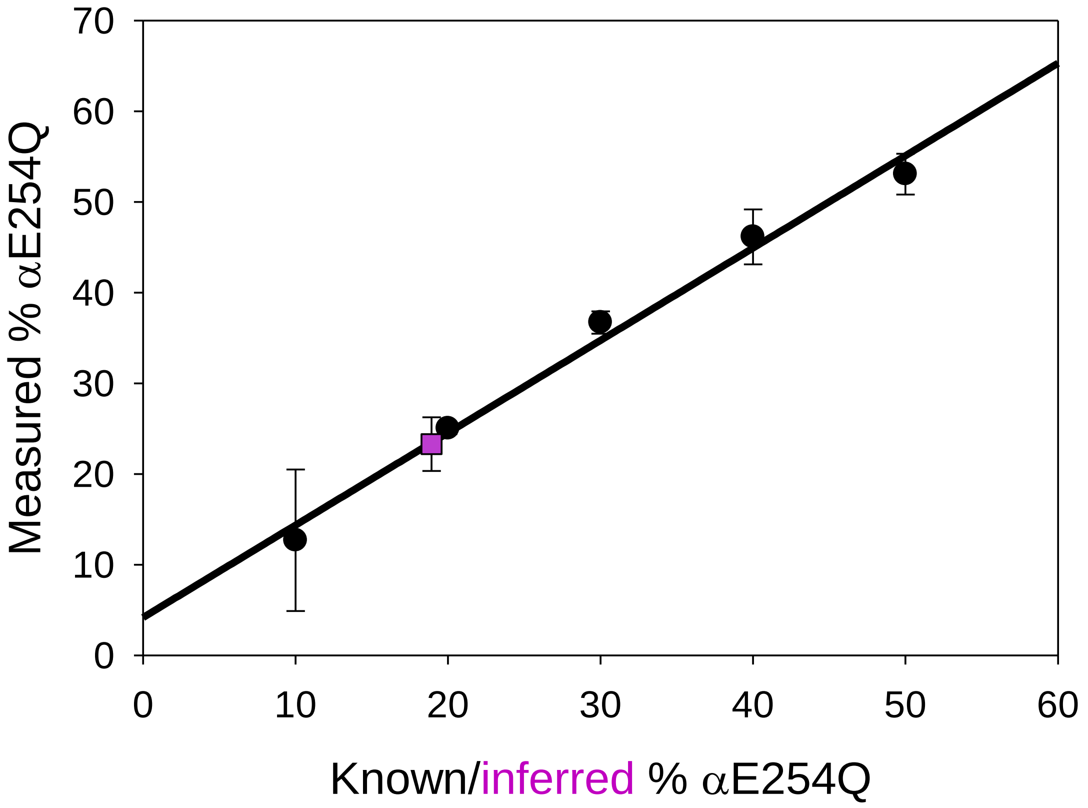

Supplementary Figure 4. LC-MS/MS mass spectrometry analysis. The measured percentages of αE254Q-tubulin in (unpolymerized) αE254Q+αE254N tubulin mixtures are plotted as a function of the known αE254Q-tubulin content of the mixtures (black circles) to obtain a calibration curve (black line). The percentage of αE254Q-tubulin in 20%Q+N-mosaic microtubules was determined from the measured percentage, corrected by the calibration curve, yielding 18.9% ± 4.3 (purple square). Data represent mean values from N = 3 independent experiments, with n = 2 replicas per condition. Error bars indicate SD.

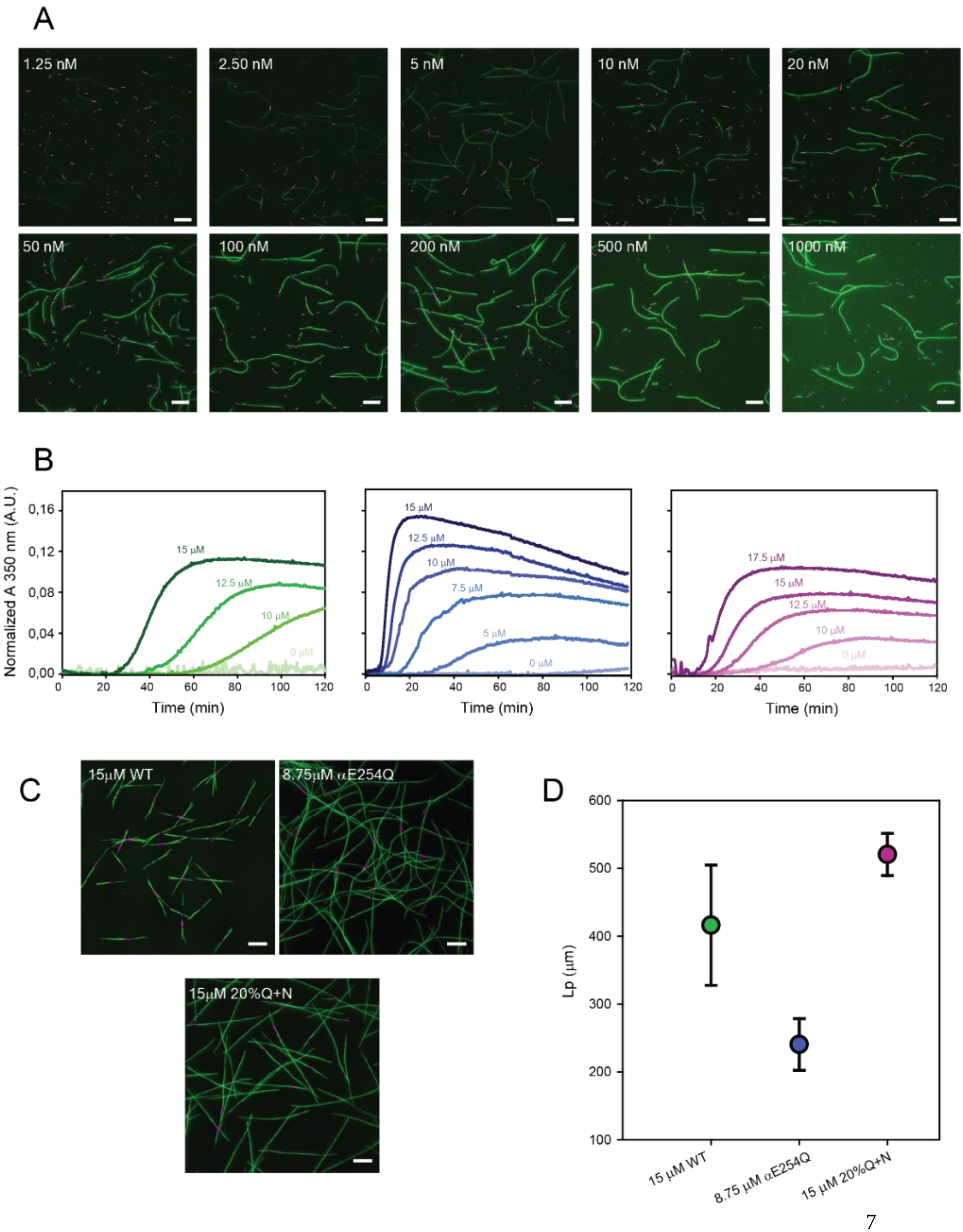
A
1.25 nM
2.50 nM
5 nM
10 nM
20 nM
50 nM
100 nM
200 nM
500 nM
1000 nM
B
Normalized A 350 nm (A.U.)
0,16
0,12
0,08
0,04
0,00
15 µM
12.5 µM
10 µM
0 µM
Time (min)
15 µM
12.5 µM
10 µM
7.5 µM
5 µM
0 µM
Time (min)
17.5 µM
15 µM
12.5 µM
10 µM
0 µM
Time (min)
C
15µM WT
8.75µM αE254Q
15µM 20%Q+N
D
Lp (µm)
600
500
400
300
200
100
15 µM WT
8.75 µM αE254Q
15 µM 20%Q+N

Supplementary Figure 5. Biochemical characterization of 20%Q+N-mosaic microtubules. A. TIRF microscopy images of 20%Q+N-microtubules in the presence of different mGFP-EB3 concentrations, as indicated, used to determine the Kd of mGFP-EB3 binding (Fig. 4A). **B.** Time course of turbidity of WT (left, green scale), αE254Q (center, blue scale), and 20%Q+N-microtubules (right, purple scale) nucleated in solution and polymerizing at the indicated tubulin concentrations, used to calculate the apparent nucleation constant (Fig. 4E). **C, D.** TIRF microscopy images of microtubules elongated from GMPCPP-microtubule seeds at the indicated tubulin concentrations (C), used to calculate the average persistence lengths (Lp) of WT, αE254Q, and 20%Q+N-microtubules (D). Scale bars represent 10 µm. n = 25 filaments per condition. Error bars represent SD.

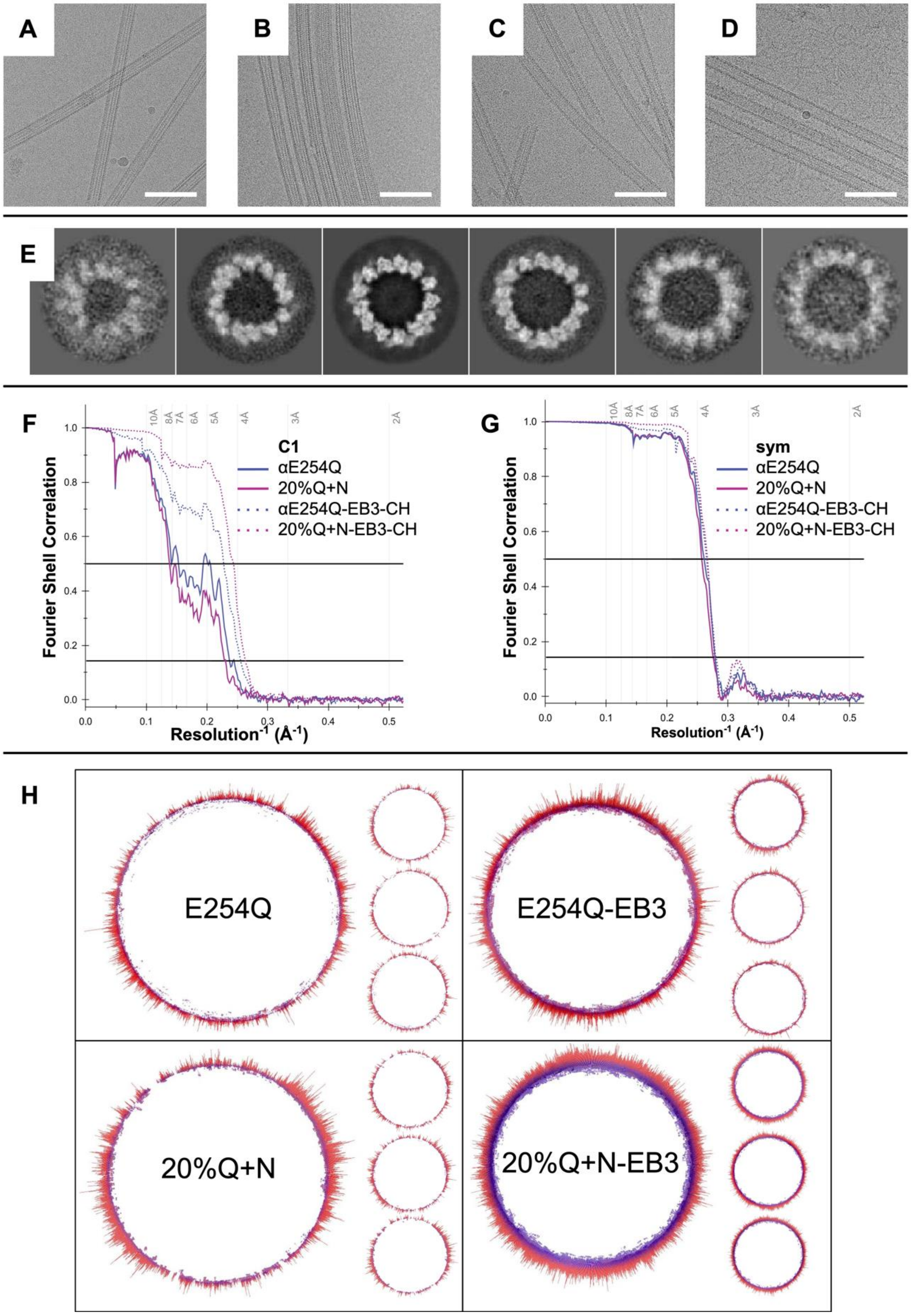
A
B
C
D
E
F
G
H
C1
αE254Q
20%Q+N
αE254Q-EB3-CH
20%Q+N-EB3-CH
sym
Fourier Shell Correlation
Resolution^{-1} (Å^{-1})
E254Q
E254Q-EB3
20%Q+N
20%Q+N-EB3

Supplementary Figure 6. Cryo-EM analysis of microtubules. A-D. Drift-corrected and dose-weighted micrographs showing microtubules of the four samples that were investigated in this study: αE254Q- (A), 20%Q+N- (B), αE254Q-EB3-CH- (C), and 20%Q+N-EB3-CH-microtubules (D). Scale bars, 100 nm. **E**. Picked microtubules were categorized into six classes during data processing. In this representative figure, the classes are derived from the data of the EB3-decorated 20% Q+N microtubules. The six classes, displayed from left to right, include 11, 12, 13, 14, 15, and 16 protofilaments. Notably, the class with 13 protofilaments consistently contained the highest number of particles, making it less noisy. **F, G.** Map:map FSC resolution plots of the final cryo-EM maps of the 13 protofilament microtubules. In panel F**,** the results of the unsymmetrized maps that have a resolution between 3.8 and 4.3 Å are shown; in panel G**,** the symmetrized maps that have a resolution of 3.6 Å are shown. **H** Representation of particle angular distribution. The images show the angular distributions of the four different datasets (large figures). The distributions show even orientations around the microtubule cross-section, forming a ring pattern. After splitting each dataset in three (see Methods for further details); the split datasets (small figures) show the same even angular distribution.

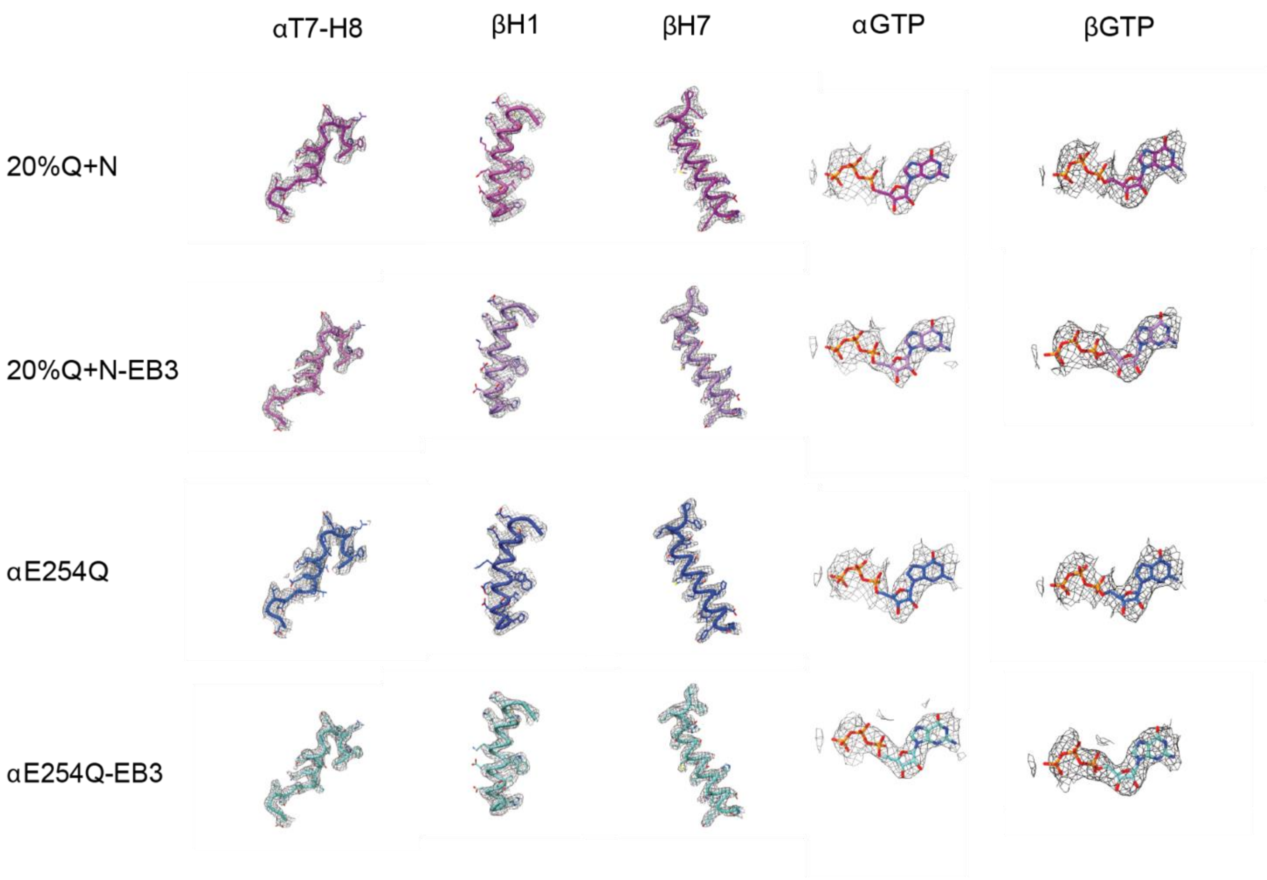

Supplementary Figure 7. Model fitting into symmetrized electron density maps. Sections of the symmetrized electron density maps illustrating the quality of model fitting in representative regions of αβ-tubulin and bound nucleotides.

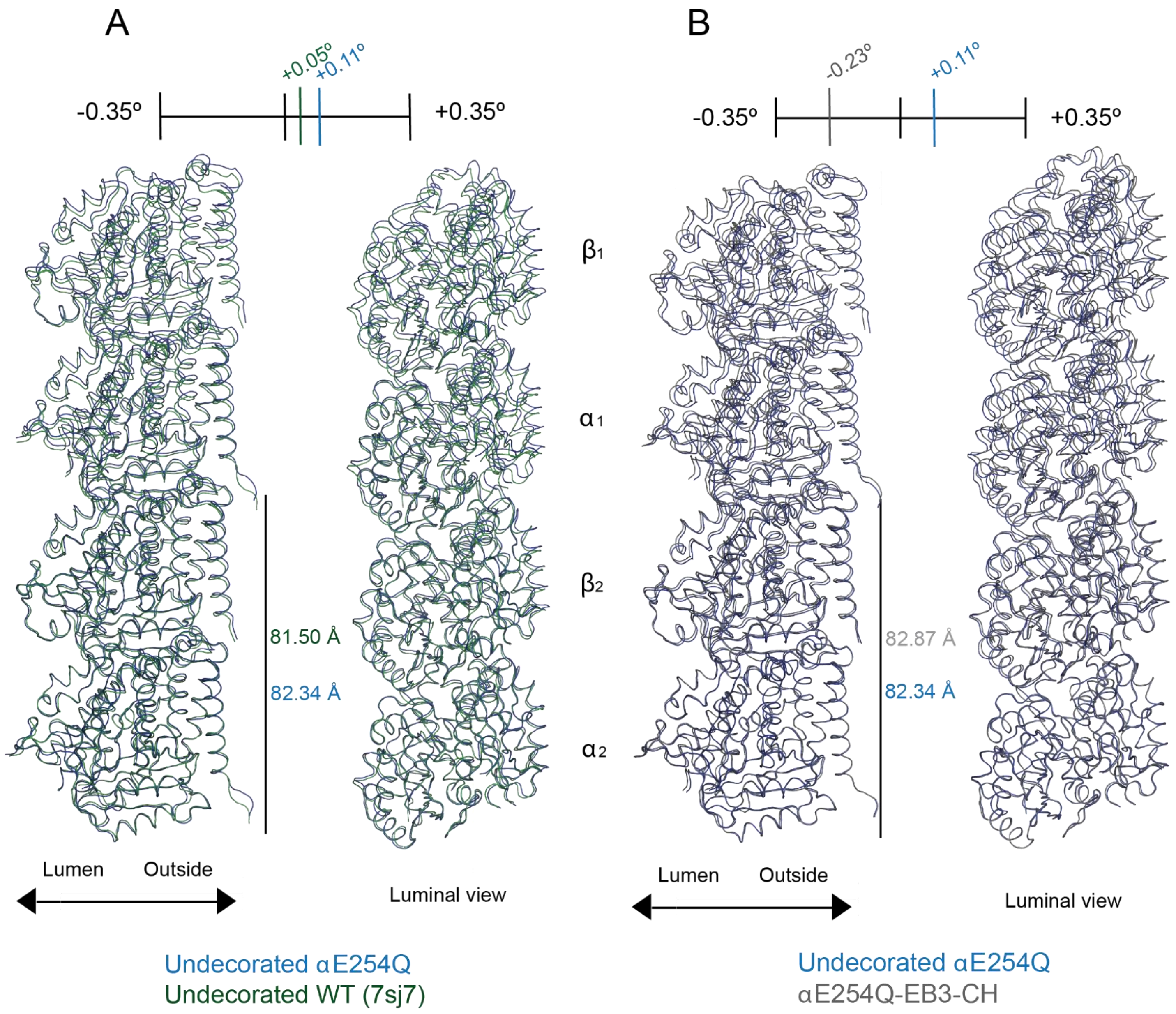

Supplementary Figure 8. **Atomic models of αE254Q-microtubules.** Atomic model building of αE254Q microtubules from their corresponding symmetrized maps. Comparison of the Cα traces of two consecutive dimers after model superimposition on the intermediate domain of the α_2-tubulin chain. **A.** Comparison between undecorated αE254Q-microtubules (blue) and αE254E microtubules (WT-microtubules, green) (PDB: 7SJ7)[34] [https://doi.org/10.2210/pdb7SJ7/pdb] **B.** Comparison between undecorated αE254Q-microtubules (blue) and EB3-CH-αE254Q-microtubules (grey). The dimer rise (next to bottom tubulin dimers) and protofilament twist (at the top) as determined from the C1 maps using RELION are indicated.

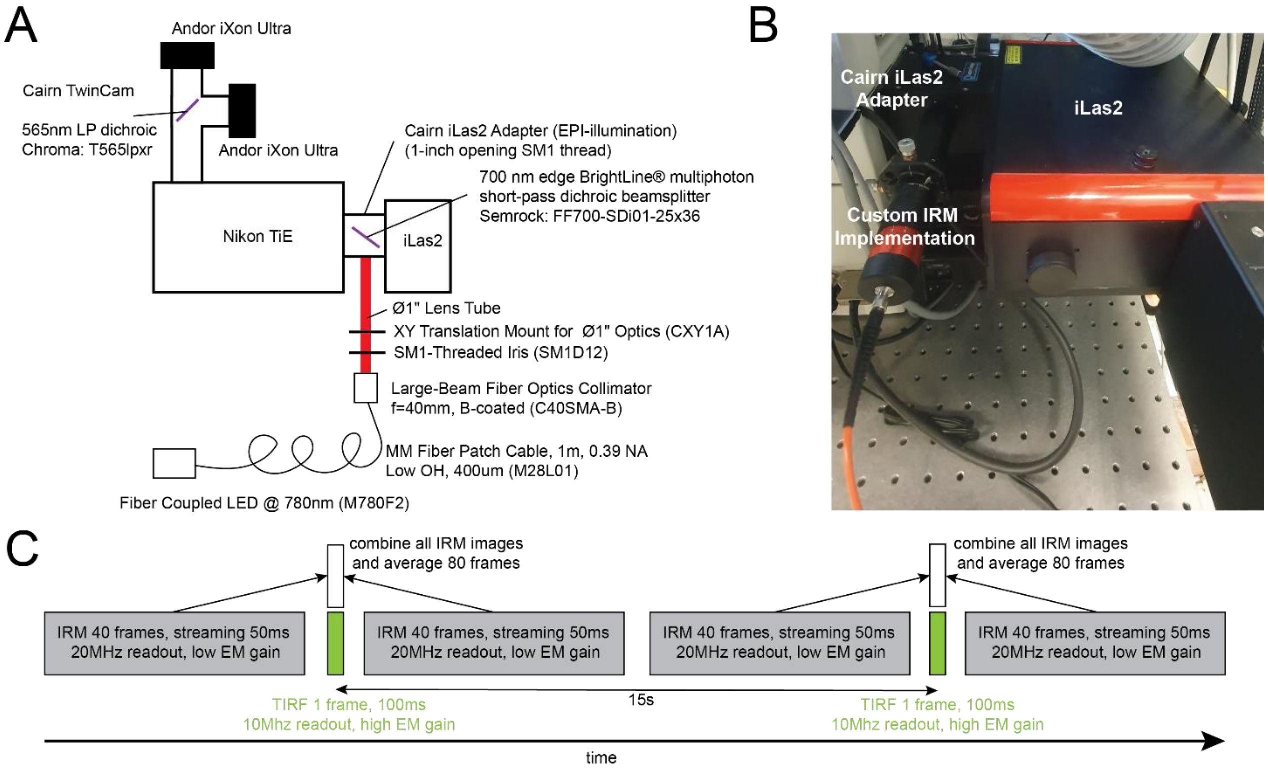

Supplementary Figure 9. Combined TIRF/IRM time-lapse imaging. A. Schematic of the IRM implementation on a dual-camera TIRF system with rotating TIRF (iLas2, Gataca Systems, France). All parts numbers are from Thorlabs (USA) unless indicated otherwise. **B**. Photo of the IRM add-on installed at the EPI-port of the Cairn iLas2 adapter. **C**. Schematic chart for time-lapse imaging. 40 IRM images were recorded in fast stream acquisition mode, 1 TIRF frame was acquired, and then again 40 IRM images. All 80 IRM images were averaged to a single frame, resulting in one TIRF and one IRM image with approximately the same acquisition time. This process was repeated yielding a time-lapse for both IRM and TIRF with a 15 s time-resolution.

Supplementary Table 1. Microtubule growth rates (nm/s, Average ± Stdev)

	**WT**	**αE254Q**	**αE254N**	**20%Q+N**
1.25 µM	-	3.4 ± 0.5	-	-
2.50 µM	-	9.0 ± 0.9	-	-
3.75 µM	-	10.4 ± 1.2	-	-
5.00 µM	-	15.6 ± 2.4	-	9.9 ± 1.5
6.25 µM	-	22.0 ± 1.7	-	-
7.50 µM	-	25.9 ± 2.5	-	16.8 ± 3.3
8.75 µM	-	32.0 ± 2.1	-	-
10.0 µM	21.1 ± 3.4	-	6.5 ± 2.6	22.9 ± 2.4
12.5 µM	24.1 ± 4.0	-	15.3 ± 2.9	28.5 ± 3.4
15.0 µM	32.7 ± 4.8	-	18.2 ± 3.2	32.7 ± 3.1
17.5 µM	37.9 ± 5.9	-	19.0 ± 3.0	38.8 ± 3.8
20.0 µM	44.6 ± 7.4	-	24.8 ± 0.3	-

Supplementary Table 2. Microtubule protofilament number relative frequencies

	**αE254Q**	**αE254Q-EB3-CH**	**αE254QN**	**αE254QN-EB3-CH**
Number of particles of 12-15 pfs	57,294	73,019	36,543	142,220
12 pf [%]	51.14	43.97	10.22	4.02
13 pf [%]	46.40	47.05	88.35	89.93
14 pf [%]	1.57	7.72	0.80	5.28
15 pf [%]	0.89	1.26	0.63	0.78

Supplementary Table 3. Cryo-EM data collection, refinement and validation statistics

	**13pf-αE254Q** (EMDB-50172) (PDB 9F3B)	**13pf-αE254Q-EB3-CH** (EMDB-50177) (PDB 9F3R)	**13pf-20%Q+N** (EMDB-50174) (PDB 9F3H)	**13pf-20%Q+N-EB3-CH** (EMDB-50178) (PDB 9F3S)
**Data collection and processing**				
Magnification	146,597 x	146,597 x	146,597 x	146,597 x
Voltage (kV)	200	200	200	200
Electron exposure (e–/Å^2)	7.25	7.19	7.25	7.27
Defocus range (μm)	-1.0 to -2.5	-0.7 to -2.2	-1.0 to -2.5	-1.0 to -2.5
Pixel size (Å)	0.955	0.955	0.955	0.955
Symmetry imposed	Helical	Helical	Helical	Helical
Initial particle images	71,850	79,270	44,726	163,854
Final 13PFs particle images	26,038	33,410	30,691	142,220
C1 Map resolution (Å)	4.2	3.9	4.3	3.8
Sym Map resolution (Å)	3.6	3.6	3.6	3.6
**Refinement**				
Initial model used (PDB code)	7sj7	7sj7	7sj7	7sj7
Model resolution (Å)	3.85	3.90	3.80	3.80
$FSC_{model\ (0/0.143/0.5)}$	3.4/3.5/3.9	3.4/3.5/3.6	3.5/3.6/3.9	3.4/3.5/3.6
Non-hydrogen atoms	41,106	43,264	41,100	43,258
Protein residues	5,184	5,446	5,184	5,446
Ligands	24	24	24	24
Validation:				
Molprobity score	1.81	1.77	1.84	1.55
Clashscore, all atoms	11.83	9.92	10.74	7.53
CaBLAM outliers (%)	0.96	0.93	0.96	0.93
Poor rotamers	0.34	0.73	0.29	0.41
Ramachandran plot				
Favored (%)	96.59	96.31	95.74	97.29
Allowed (%)	3.41	3.69	4.26	2.71
Outliers (%)	0.00	0.00	0.00	0.00
