# Supplementary material for: Hydrolysis-deficient mosaic microtubules as faithful mimics of the GTP cap
Source: Nat Commun. 2025 Mar 10;16:2396. doi: 10.1038/s41467-025-57555-6 (PMC11893814; doi:10.1038/s41467-025-57555-6)
Supplement: Supplementary file 3 — Reporting Summary [file 41467_2025_57555_MOESM3_ESM.pdf]

nature portfolio

Corresponding author(s): Estévez-Gallego, J.
Surrey, T.

Last updated by author(s): Feb 2, 2025

Reporting Summary

Nature Portfolio wishes to improve the reproducibility of the work that we publish. This form provides structure for consistency and transparency in reporting. For further information on Nature Portfolio policies, see our Editorial Policies and the Editorial Policy Checklist.

Statistics

For all statistical analyses, confirm that the following items are present in the figure legend, table legend, main text, or Methods section.

n/a	Confirmed	
☐	☒	The exact sample size (*n*) for each experimental group/condition, given as a discrete number and unit of measurement
☐	☒	A statement on whether measurements were taken from distinct samples or whether the same sample was measured repeatedly
☐	☒	The statistical test(s) used AND whether they are one- or two-sided<br>*Only common tests should be described solely by name; describe more complex techniques in the Methods section.*
☒	☐	A description of all covariates tested
☒	☐	A description of any assumptions or corrections, such as tests of normality and adjustment for multiple comparisons
☐	☒	A full description of the statistical parameters including central tendency (e.g. means) or other basic estimates (e.g. regression coefficient) AND variation (e.g. standard deviation) or associated estimates of uncertainty (e.g. confidence intervals)
☒	☐	For null hypothesis testing, the test statistic (e.g. *F*, *t*, *r*) with confidence intervals, effect sizes, degrees of freedom and *P* value noted<br>*Give P values as exact values whenever suitable.*
☒	☐	For Bayesian analysis, information on the choice of priors and Markov chain Monte Carlo settings
☒	☐	For hierarchical and complex designs, identification of the appropriate level for tests and full reporting of outcomes
☒	☐	Estimates of effect sizes (e.g. Cohen's *d*, Pearson's *r*), indicating how they were calculated

Our web collection on statistics for biologists contains articles on many of the points above.

Software and code

Policy information about availability of computer code

Data collection	MetaMorph, Proteome Discoverer software suite (v2.5, Thermo Fisher Scientific), Mascot search engine (v2.6, Matrix Science), Skyline-Daily 22.2.1.352, EPU3 software.
Data analysis	Image J 1.53t, JASP 0.16.3, MATLAB, FIESTA, SigmaPlot 11.0, Proteome Discover 2.5, Mascot search engine 2.6, Relion 3.1.2 , MotionCor2, Gctf, Coot 0.9.8, Phenix 1.21, UCSF ChimeraX 1.8, PyMol 1.2r3pre.

For manuscripts utilizing custom algorithms or software that are central to the research but not yet described in published literature, software must be made available to editors and reviewers. We strongly encourage code deposition in a community repository (e.g. GitHub). See the Nature Portfolio guidelines for submitting code & software for further information.

Data

Policy information about availability of data

All manuscripts must include a data availability statement. This statement should provide the following information, where applicable:

- Accession codes, unique identifiers, or web links for publicly available datasets
- A description of any restrictions on data availability
- For clinical datasets or third party data, please ensure that the statement adheres to our policy

The authors declare that the data supporting the findings of this study are available within the paper,its supplementary information files and in the provided source data file. Atomic coordinates of the symmetrized models have been deposited in the Protein Data Bank (PDB) with accession numbers 9F3B [https://

April 2023

doi.org/10.2210/pdb9F3B/pdb] (undecorated 13-protofilament αE254Q microtubules), 9F3H [https://doi.org/10.2210/pdb9F3H/pdb] (undecorated 13-protofilament 20%Q+N microtubules), 9F3R [https://doi.org/10.2210/pdb9F3R/pdb] (EB3-decorated 13-protofilament αE254Q microtubules) and ,9F3S [https://doi.org/10.2210/pdb9F3S/pdb] (EB3-decorated 13-protofilament 20%Q+N microtubules). The corresponding cryo-EM density maps have been deposited in the Electron Microscopy Data Bank under the accession numbers EMD-50172 [https://www.ebi.ac.uk/pdbe/entry/emdb/EMD-50172] (undecorated 13-protofilament αE254Q microtubules), EMD-50174 [https://www.ebi.ac.uk/pdbe/entry/emdb/EMD-50174] (undecorated 13-protofilament 20%Q+N microtubules), EMD-50177 [https://www.ebi.ac.uk/pdbe/entry/emdb/EMD-50177] (EB3-decorated 13-protofilament αE254Q microtubules) and EMD-50178 [https://www.ebi.ac.uk/pdbe/entry/emdb/EMD-50178] (EB3-decorated 13-protofilament 20%Q+N microtubules). The mass spectrometry proteomics data have been deposited to the ProteomeXchange Consortium via the PRIDE 71 partner repository with the dataset identifier PXD058083

Research involving human participants, their data, or biological material

Policy information about studies with human participants or human data. See also policy information about sex, gender (identity/presentation), and sexual orientation and race, ethnicity and racism.

Reporting on sex and gender	n/a
Reporting on race, ethnicity, or other socially relevant groupings	n/a
Population characteristics	n/a
Recruitment	n/a
Ethics oversight	n/a

Note that full information on the approval of the study protocol must also be provided in the manuscript.

Field-specific reporting

Please select the one below that is the best fit for your research. If you are not sure, read the appropriate sections before making your selection.

☒ Life sciences ☐ Behavioural & social sciences ☐ Ecological, evolutionary & environmental sciences

For a reference copy of the document with all sections, see nature.com/documents/nr-reporting-summary-flat.pdf

Life sciences study design

All studies must disclose on these points even when the disclosure is negative.

Sample size	All experiments were independenlty repeated at least two times. No statistical methods were employed to determine sample sizes. Sample size was chosen based on previous experience and standards in the field.
Data exclusions	No data were excluded
Replication	All experiments were independently repeated at least two times. All attempts at replication were successful.
Randomization	No randomization applies for this work
Blinding	No blinding was implemented for this work

Reporting for specific materials, systems and methods

We require information from authors about some types of materials, experimental systems and methods used in many studies. Here, indicate whether each material, system or method listed is relevant to your study. If you are not sure if a list item applies to your research, read the appropriate section before selecting a response.

Materials & experimental systems

n/a	Involved in the study
☒	☐ Antibodies
☐	☒ Eukaryotic cell lines
☒	☐ Palaeontology and archaeology
☒	☐ Animals and other organisms
☒	☐ Clinical data
☒	☐ Dual use research of concern
☒	☐ Plants

Methods

n/a	Involved in the study
☒	☐ ChIP-seq
☒	☐ Flow cytometry
☒	☐ MRI-based neuroimaging

Eukaryotic cell lines

Policy information about cell lines and Sex and Gender in Research

Cell line source(s)	Trichoplusia Ni HighFive cells (B85502, Gibco) and Spodoptera frugiperda Sf21(R) cells (12682019, Gibco)
Authentication	Authenticated by manufacturer
Mycoplasma contamination	All cell lines employed for protein expression were tested negative for Mycoplasma contamination
Commonly misidentified lines (See ICLAC register)	N/A

Plants

Seed stocks	n/a
Novel plant genotypes	n/a
Authentication	n/a
